# Effects of Low-dose Propofol or Ketamine on Coughing at Emergence from Anesthesia in Children Undergoing Tonsillectomy

**DOI:** 10.7759/cureus.7842

**Published:** 2020-04-26

**Authors:** Mohammad Ali Sahmeddini, Ashkan Panah, Alireza Ghanbari

**Affiliations:** 1 Anesthesiology, Shiraz Anesthesiology and Critical Care Research Center, Shiraz University of Medical Sciences, Shiraz, IRN

**Keywords:** tonsillectomy, ketamine, propofol, cough, anesthesia recovery period, children

## Abstract

Introduction

Coughing is commonly observed during emergence from general anesthesia. In children, smooth emergence from anesthesia, especially after tonsillectomy, is crucial. In this study, we compared the effect of low-dose ketamine or propofol on emergence coughing in children undergoing tonsillectomy.

Methods

In this randomized clinical trial, 90 children undergoing tonsillectomy were randomly allocated into two groups: children in group A received 0.5-mg/kg propofol and children in group B received 0.5-mg/kg ketamine, at the end of anesthesia. The incidence and severity of cough, postoperative sedation, nausea, and vomiting, and pain score were recorded and compared.

Results

The incidence of no cough at emergence from anesthesia was 82.2% in the propofol group and 15.5% in the ketamine group (P = 0.00). Children in the ketamine group exhibited postoperative pain but were more sedated compared with those in the propofol group (P > 0.05). The incidence of postoperative nausea and vomiting was lower in the propofol group (P < 0.05).

Conclusions

At the end of general anesthesia with isoflurane in children undergoing tonsillectomy, 0.5-mg/kg propofol is more effective than 0.5-mg/kg ketamine in reducing cough response upon emergence from anesthesia, with a lower incidence of nausea and vomiting, as well as lower sedation in children.

## Introduction

In children, smooth emergence from anesthesia, especially after tonsillectomy, is crucial [1]. At the end of surgery, an anesthesiologist usually decreases the depth of anesthesia to wake the child, but the endotracheal tube (ETT) can act as a foreign body and cause both cough and straining during emergence [2,3]. However, coughing and straining during emergence following tonsillectomy may cause post-tonsillectomy bleeding and laryngospasm, and increase pain and agitation [4].

Several studies have reported various methods and drugs to reduce the incidence of coughing and straining during emergence from anesthesia [5,6]. These include extubation at the deep plane of anesthesia, use of reinforced laryngeal mask instead of an ETT, and drugs such as intravenous or intratracheal tube lidocaine, intravenous magnesium sulfate, ketamine, and opioids [7-11]. Recent studies have highlighted the possible role of low-dose propofol as a suppressant of airway reflexes in noninvasive operations [12].

However, no studies have compared the effect of propofol with ketamine in reducing coughing and straining after tonsillectomy. Therefore, this study was conducted to compare the effect of ketamine to propofol in reducing coughing at emergence from anesthesia in children who underwent tonsillectomy.

## Materials and methods

This parallel, double-blind, randomized clinical trial was conducted in a tertiary hospital of Shiraz University of Medical Sciences. After receiving approval from the ethics committee of Shiraz University of Medical Sciences, the trial was registered at the Islamic Republic of Iran Clinical Trials (IRCT) registry (registration number 2016101411662N11). Overall, 90 children aged 3-12 years in the ASA (American Society of Anesthesiologists) class I or II who were scheduled to undergo elective tonsillectomy under general anesthesia (G/A) were enrolled in this study. Children with a history of obstructive sleep apnea syndrome, bronchial asthma, allergic disorders, and upper respiratory tract infection symptoms prior to surgery were excluded from the study. Furthermore, those who use angiotensin-converting enzyme (ACE) inhibitors, developmental mental disorders, airway or facial abnormalities, and in whom the anesthesiologist tried more than once for endotracheal intubation were excluded from the study. The study protocol was explained to parents of the eligible children, and written informed consent was obtained from parents. The eligible children were randomly assigned into two groups (A and B) through simple randomization using computer-generated random numbers. Children in group A received propofol and those in group B received ketamine, at the end of anesthesia. This randomization was performed by a nurse anesthetist who had no role in administering the study.

In the operating room, routine monitoring for each child included oxygen saturation (SpO_^2^_), electrocardiogram, noninvasive blood pressure, and end-tidal carbon dioxide (EtCO_2_). Induction of anesthesia was similar in both groups, in­cluding midazolam (0.03 mg/kg), fentanyl (2 µg/kg), thiopental (5 mg/kg), and atracurium (0.6 mg/kg). Tracheal intubation with a suitable size was performed by an expert anesthesiologist in a single attempt. Anesthesia was maintained with 1.2% isoflurane in N_2_O/O_2_ (50%/50%) using controlled ventilation to maintain EtCO_2_ between 35 and 40 mmHg. At the end of the operation, isoflurane and N_2_O were discontinued and 100% oxygen was administered, and when the children’s breathing returned to the spontaneous pattern, the residual neuromuscular block was reversed by neostigmine (0.04 mg/kg) plus atropine (0.015 mg/kg). After regular spontaneous breathing, children in groups A and B intravenously received 0.5-mg/kg propofol (Provive 1%, Claris Lifesciences Ltd., Ahmedabad, Gujarat, India) and 0.5-mg/kg ketamine, respectively (Rotexmedica, Trittau, Germany). The dose of propofol and ketamine were selected according to Ozturk et al [13]. The ETT was removed after spontaneous breathing with an adequate tidal volume, and EtCO2 was achieved. After extubation, the children were transferred to the post-anesthesia care unit (PACU), given 5-6 L/minute of humidified oxygen through a facemask, and monitored for SpO_2_ and heart rate.

The primary outcome of this study was the incidence of cough at emergence, which was evaluated by cough scores. Cough scores were recorded based on the number of coughs: 0 (no coughing), 1 (minimal: once or twice), 2 (moderate: three to four times), or 3 (severe: five or more times). The scores were recorded when the children were extubated (time 0), every five minutes thereafter, and until 30 minutes after operation.

**Figure 1:**
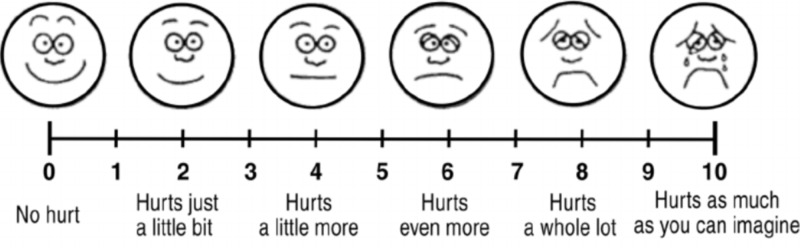
Pain Faces Scale - Revised

The secondary outcomes of this study were postoperative pain, sedation, and nausea and vomiting. Postoperative pain was measured by the Pain Faces Scale - Revised (Figure 1) in PACU every 10 minutes until 30 minutes and then at 1, 2, 4, 8, and 12 hours postoperation. Children with a score of 6/10 or higher were considered having moderate-to-severe pain and were treated with 0.2 mg/kg of intravenous meperidine.

Postoperative sedation score was recorded according to the Ramsay Sedation Score (0 = restless, 1 = calm, 2 = sleepy, 3 = drowsy with response to verbal stimuli, 4 = drowsy without response to verbal stimuli, and 5 = deep without response to painful stimuli) [14]. Postoperative nausea and vomiting were recorded as 0 = no nausea or vomiting, 1 = nausea only, and 2 = retching and/or vomiting.

Children were evaluated during the study by a researcher who was not aware of patient group allocation.

 Statistical analysis

According to previous studies, the primary endpoint of the study was the incidence of cough at emergence from anesthesia [12,13]. Therefore, for a 20% difference in the incidence of cough at emergence between the two groups and with a power of 80% and α-error being set at 0.05, at least 45 patients were required in each group. Initially, the Kolmogorov-Smirnov test was used to detect the normal distribution of the variables. Then, mean and standard deviation (SD) were evaluated for numerical variables and frequency with percentage for categorical variables. For analysis of parametric data, independent t-tests and one-way ANOVA (analysis of variance) were used, and for comparison of nonparametric data, χ^2^ tests were used. All statistical analyses were performed using SPSS Version 18 (SPSS Inc., Chicago, IL, USA). All data are presented as means ± SD and percentage, and a p-value of <0.05 was considered statistically significant.

## Results

**Figure 2:**
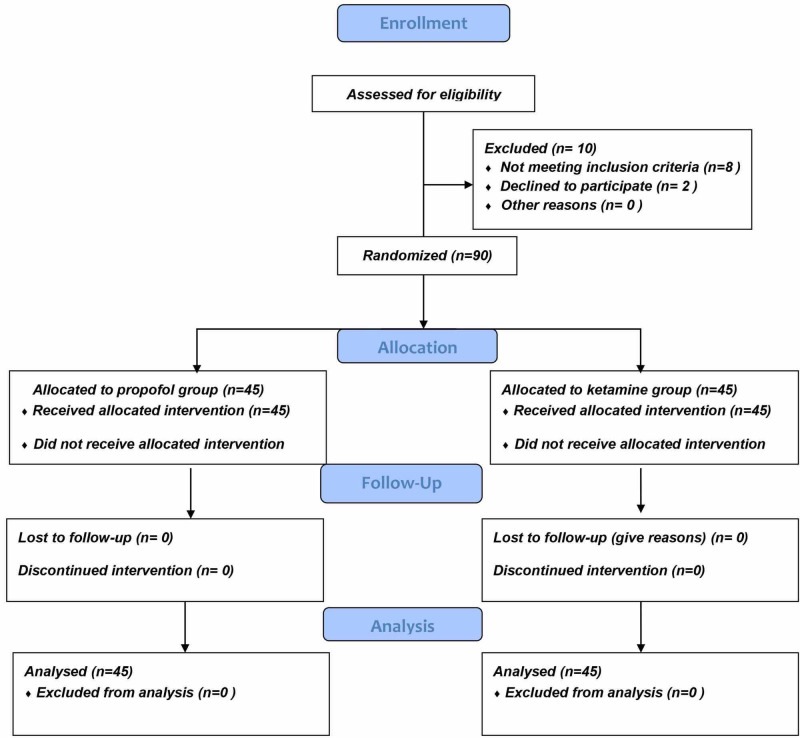
Flowchart of the patients according to the CONSORT guidelines. CONSORT, Consolidated Standards of Reporting Trials

Among the 100 children who were scheduled to undergo tonsillectomy from August 2017 to October 2017, 5 were excluded because of acute respiratory tract infection, 3 due to a history of bronchial asthma, and 2 due to parental refusal. Therefore, in total 90 children were enrolled for the study and randomly allocated to groups A and B (Figure 2).

There were no significant differences in the demographic data, duration of anesthesia, time to extubation, and total fluid received by each participant (P > 0.05; Table 1).

**Table 1:** Patients demographic data, anesthesia time, extubation time and fluid therapy between the two groups Data are expressed as mean ± standard deviation or as number (proportion).

	Propofol group (n=45)	Ketamine group (n=45)	P-value
Age (years)	6.64 ± 2.54	6.17 ± 2.07	0.333
Weight (kg)	23.96 ± 9.99	22.66 ± 6.86	0.471
Height (cm)	110.42 ± 15.74	104.62 ± 20.18	0.130
Gender (male/female)	29/16	30/15	1.133
Duration of anesthesia (minutes)	41.08 ± 2.99	40.64 ± 2.75	0.460
Time to extubation (minutes)	5.7 ± 3.02	7.2 ± 0.43	0.510
Fluid replacement (mL)	304.11 ± 115.02	300.22 ± 81.11	0.850

The incidence of no cough at emergence from anesthesia was 82.2% in the propofol group and 15.5% in the ketamine group (P = 0.00).The number of children who developed mild-to-moderate cough upon emergence and during 30 minutes after that is shown in Table 2. No participant developed severe cough on emergence from anesthesia and 30 minutes after that in both groups (Table 2).

**Table 2:** Number of patients who developed cough at emergence and 30 minutes after that in both groups

	Propofol group (n=45)				Ketamine group (n=45)				
Time	No cough	Minimal cough	Moderate cough	Severe cough	No cough	Minimal cough	Moderate cough	Severe cough	P-value
0 minutes	37	8	0	0	7	24	14	0	0.000
5 minutes	44	1	0	0	10	19	16	0	0.001
10 minutes	42	3	0	0	15	20	10	0	0.009
15 minutes	43	2	0	0	18	21	6	0	0.001
20 minutes	36	9	0	0	20	15	10	0	0.003
30 minutes	38	7	0	0	18	19	8	0	0.002

Children in the ketamine group had lower postoperative pain score than those in the propofol group until two hours postoperation (P = 0.001; Table 3); after that, there were no significant differences in pain scores of both groups due to pain rescue drug (P > 0.05; Table 3).

**Table 3:** Postoperative pain score among children in the two groups according to the Pain Faces Scale - Revised

Time	Ketamine group (n=45)	Propofol group (n=45)	P-value
10 minutes	1.88 ± 0.44	8.26 ± 0.49	0.008
20 minutes	2.22 ± 0.59	6.64 ± 1.88	0.005
30 minutes	2.73 ± 0.98	4.24 ± 0.77	0.007
60 minutes	2.33 ± 0.52	4.62 ± 2.83	0.001
2 hours	1.08 ± 1.44	2.91 ± 0.92	0.58
4 hours	0.08 ± 0.44	0.24 ± 0.44	0.515
8 hours	0.00 ± 00	0.00 ± 00	0.517
12 hours	0.00 ± 00	0.00 ± 00	0.22

Furthermore, children in the propofol group were more calm and less drowsy than those in the ketamine group (P < 0.05; Table 4).

**Table 4:** Postoperative agitation scores of children in both groups

Sedation score	Ketamine group (n=45)	Propofol group (n=45)	P-value
Restless	1 (2.22%)	5 (11.11%)	0.01
Calm	15 (33.33%)	35 (77.77%)	0.001
Sleepy	8 (17.77%)	4 (8.88%)	0.01
Drowsy with response to verbal stimuli	16 (35.55%)	1 (2.22%)	0.001
Drowsy without response to verbal stimuli	5 (11.11%)	0 (0.00%)	0.01
Deep without response to painful stimuli	0 (0.00%)	0 (0.00%)	0.98

**Figure 3:**
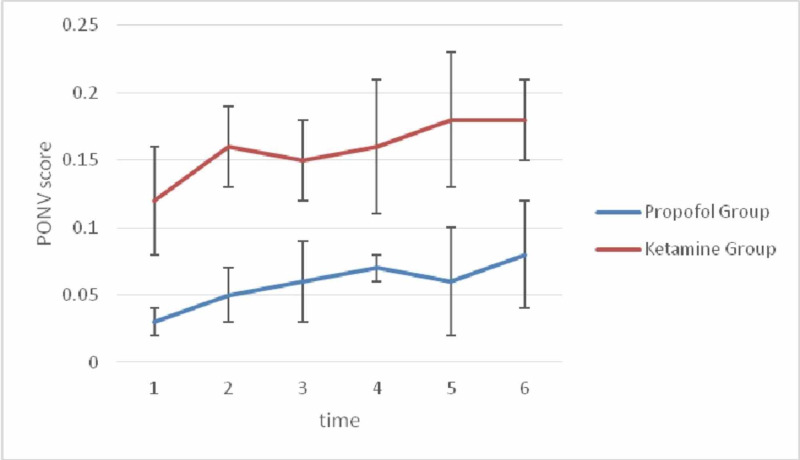
PONV in both groups during six hours postoperation. PONV, postoperative nausea and vomiting

Significant differences were also observed in the incidences of nausea and vomiting between the two groups during six hours postoperation (P = 0.002; Figure 3).

## Discussion

This randomized clinical trial showed that a low dose of propofol (0.5 mg/kg) compared with a low dose of ketamine was more effective in reducing cough at emergence from isoflurane/N2O anesthesia in children who underwent tonsillectomy.

Coughing and their afferent fibers in the vagus nerve at emergence from anesthesia is a reflex that is due to chemical or mechanical stimulation of tissue nerve ending [15]. Also, cough reflex may be induced by other nerves and organs such as the trachea, diaphragm, and abdominal muscle [16]. Stimulation of N-methyl-D-aspartate (NMDA) receptors in the trachea causes coughing response by ascending pathway from the trachea. Therefore, NMDA receptors play a key role in coughing response [17].

Previous studies have shown that ketamine effectively suppresses NMDA receptors, relaxes the smooth muscle of bronchus, and prevents bronchoconstriction [1]. Therefore, ketamine has been used as a bronchodilator by anesthesiologists [18]. Recently, ketamine has been shown to reduce laryngospasm, but not coughing, at emergence from anesthesia [12]. In this study, we also found ketamine to be not as effective as propofol in reducing the incidence of cough at emergence from anesthesia.

A study by Jung et al. showed that propofol could reduce cough response at emergence from anesthesia [19]. Another study by Orser et al. reported that propofol could effectively inhibit NMDA receptors. Therefore, propofol could diminish cough reflex due to airway irritation by blocking the NMDA receptors in the trachea and blocking the ascending pathway from the trachea [20]. Although ketamine is an NMDA receptor blocker, Pak et al. showed that low-dose ketamine could not diminish cough response upon emergence from anesthesia in children, possibly because ketamine increases bronchotracheal secretion that induces cough response, hence resulting in the negative effect of ketamine on the cough upon emergence from anesthesia [12].

Our results were similar to those by Pak et al., and propofol was found more effective than ketamine in reducing cough response upon emergence from anesthesia [12]. However, we used isoflurane, which is more irritating to the airway than sevoflurane, which was used by Pak et al. Furthermore, all the children in our study underwent tonsillectomy, which is considered an invasive throat surgery that raises the risk of cough response. However, the study by Pak et al. included children who underwent herniorraphy, and the upper airway was not manipulated. Therefore, our study has stronger evidence to support the antitussive effect of low-dose propofol in children.

Another study by Ozturk et al. reported that neither propofol nor ketamine was effective in decreasing the incidence of cough at emergence from anesthesia and in the postanesthesia recovery time [13]. However, they used remifentanil during anesthesia for bronchoscopy. Although opioids have a central antitussive effect, potent and short-acting opioids such as remifentanil could induce cough response through activation of the vagus nerve, and bronchoconstrictive effect. Therefore, the study did not conclude that propofol has an antitussive effect.

Similar to previous studies, our study also observed that children in the propofol group had a lower incidence of postoperative nausea and vomiting. Also, children in the propofol group had lower sedation scores and less agitation than those in the ketamine group. Children who received ketamine were more sedated in PACU, as reported previously, although we used a low dose of ketamine [12].

This study has some limitations. First, we should have another group of children as a control group. Second, in future studies, it is better to compare different doses of propofol (0.25 mg/Kg, 0.5 mg/Kg) to the find best dosage of propofol or try a mixture of low-dose ketamine and propofol (ketofol) to find the best drug mixture to decrease postoperative pain and suppress cough reflex following tonsillectomy.

## Conclusions

In conclusion, for the children who underwent tonsillectomy at the end of G/A, low-dose propofol at 0.5 mg/kg is more effective than low-dose ketamine in reducing cough at emergence from anesthesia, with a lower incidence of nausea and vomiting. Also, low-dose propofol results in a lower sedation score in children.
